# Disrupted specialist outpatient services and alternative modes of service for patients with diabetes mellitus: A population‐based, retrospective cohort study in Hong Kong

**DOI:** 10.1111/dom.70069

**Published:** 2025-08-28

**Authors:** Yuk Kam Yau, Meijiao Li, Jianchao Quan, Karen Ann Grépin, Gary Kui Kai Lau, Ivy Lynn Mak, Chak Sing Lau, Ian Chi Kei Wong, David Vai Kiong Chao, Welchie Wai Kit Ko, Cindy Lo Kuen Lam, Eric Yuk Fai Wan

**Affiliations:** ^1^ Department of Family Medicine and Primary Care, School of Clinical Medicine, Li Ka Shing Faculty of Medicine The University of Hong Kong Hong Kong China; ^2^ School of Public Health, Li Ka Shing Faculty of Medicine The University of Hong Kong Hong Kong China; ^3^ Department of Medicine, School of Clinical Medicine, Li Ka Shing Faculty of Medicine The University of Hong Kong Hong Kong China; ^4^ Department of Pharmacology and Pharmacy, Li Ka Shing Faculty of Medicine The University of Hong Kong Hong Kong China; ^5^ Aston Pharmacy School Aston University Birmingham UK; ^6^ Advanced Data Analytics for Medical Science Limited Hong Kong China; ^7^ Department of Family Medicine and Primary Health Care United Christian Hospital, Kowloon East Cluster, Hospital Authority Hong Kong China; ^8^ Department of Family Medicine and Primary Healthcare Hong Kong West Cluster, Hospital Authority Hong Kong China; ^9^ Department of Family Medicine The University of Hong Kong‐Shenzhen Hospital Shenzhen China; ^10^ The Institute of Cardiovascular Science and Medicine, Li Ka Shing Faculty of Medicine The University of Hong Kong Hong Kong China

**Keywords:** cardiovascular diseases, diabetes mellitus, mortality, primary care clinic, specialist outpatient clinics

## Abstract

**Introduction:**

Reduced specialist outpatient clinic (SOPC) services during COVID‐19 waves may have increased the risk of mortality and complications in patients with diabetes mellitus (DM). Evidence on whether alternative modes of service have mitigated these risks is lacking.

**Methods:**

In this population‐based, retrospective cohort study in Hong Kong, we included patients with DM between 2010 and 2019 and had internal medicine SOPC visits in 2019. We identified modes of care (SOPC, primary care clinics and drug‐refill service) of these patients in 2020 and followed them from January 2021 to June 2024. Cox proportional hazards models were used to estimate risks of all‐cause mortality and complications.

**Results:**

Among 96 372 patients, SOPC follow‐up disruptions in 2020 were associated with a higher risk of all‐cause mortality (hazard ratio [HR] 1.20, 95% CI 1.12–1.29) and cardiovascular diseases (CVD) incidence (HR 1.12, 95% CI 1.05–1.20). Among those affected by SOPC follow‐up disruptions in 2020, patients who had attended primary care clinics in 2020 had a lower risk of all‐cause mortality (HR 0.72, 95% CI 0.60–0.87) and CVD (HR 0.84, 95% CI 0.72–0.99) than those without any primary care clinic attendance visits.

**Conclusion:**

SOPC service disruption was associated with increased risk of mortality and CVD among patients with DM. Primary care clinic service could be a beneficial alternative mode of service to attenuate the excessive risk. These findings support a policy that follow‐up at primary care clinics should be provided for patients with DM when SOPC service is disrupted in future public health crises.

## 1 INTRODUCTION

Diabetes mellitus (DM) is a common non‐communicable disease (NCD) and has become a major public health challenge, affecting 463 million people worldwide.^1^ DM is associated with greater risks of cardiovascular diseases (CVD) and premature mortality. To reduce or delay the development of complications or mortality, international clinical practice guidelines recommend individuals with DM have a comprehensive medical evaluation at least annually, including medication adjustment, laboratory tests, and assessments of complications.^2^, ^3^, ^4^ In Hong Kong, people with DM in stable or mild conditions are managed by primary care, while complicated or severe cases are referred to specialist services.^3^, ^5^ A DM care report conducted by the Hong Kong Hospital Authority (HA), a statutory body that provides all the public health care in Hong Kong, showed that 36.7% of the patients with DM were under care in specialist services in 2019–2020.^6^

Visits to routine medical care for patients with DM were affected globally during the coronavirus disease 2019 (COVID‐19) pandemic.^7^ In Hong Kong, publicly provided specialist outpatient clinic (SOPC) services were reduced after the first confirmed local COVID‐19 case to focus the manpower and healthcare resources on the challenge of the pandemic.^8^ There are concerns that disruptions to SOPC follow‐up in patients with DM may have led to suboptimal glycaemic control and other unmet medical needs, consequently increasing risks of CVD and mortality.

Primary care services were largely maintained during the COVID‐19 pandemic in Hong Kong, which served as alternative modes of care for SOPC patients with DM. A meta‐analysis showed that structured care in primary care for people with DM is a good substitute and achieves metabolic control similar to SOPC, at least in the short term.^9^ In addition, prior studies indicated that drug‐refill service, defined as repeat dispensing of medication without clinic attendance, was associated with better glycaemic control for patients with DM than those who did not receive refills.^10^ However, it is still uncertain whether primary care clinic visits or drug‐refill services could reduce the risk of mortality and complications in patients with DM affected by SOPC disruptions during the COVID‐19 pandemic in Hong Kong.

Using a population‐based retrospective cohort of patients in Hong Kong, we aim to examine the association of disrupted SOPC service and alternative modes of care with all‐cause mortality, CVD, and kidney failure incidence among SOPC patients with DM. The findings can not only suggest a preferable approach to ongoing management of patients with DM, but also inform health policies about the contingency care plans in future public health crises.

## 2 METHODS

### 2.1 Study design and population

**FIGURE 1:**
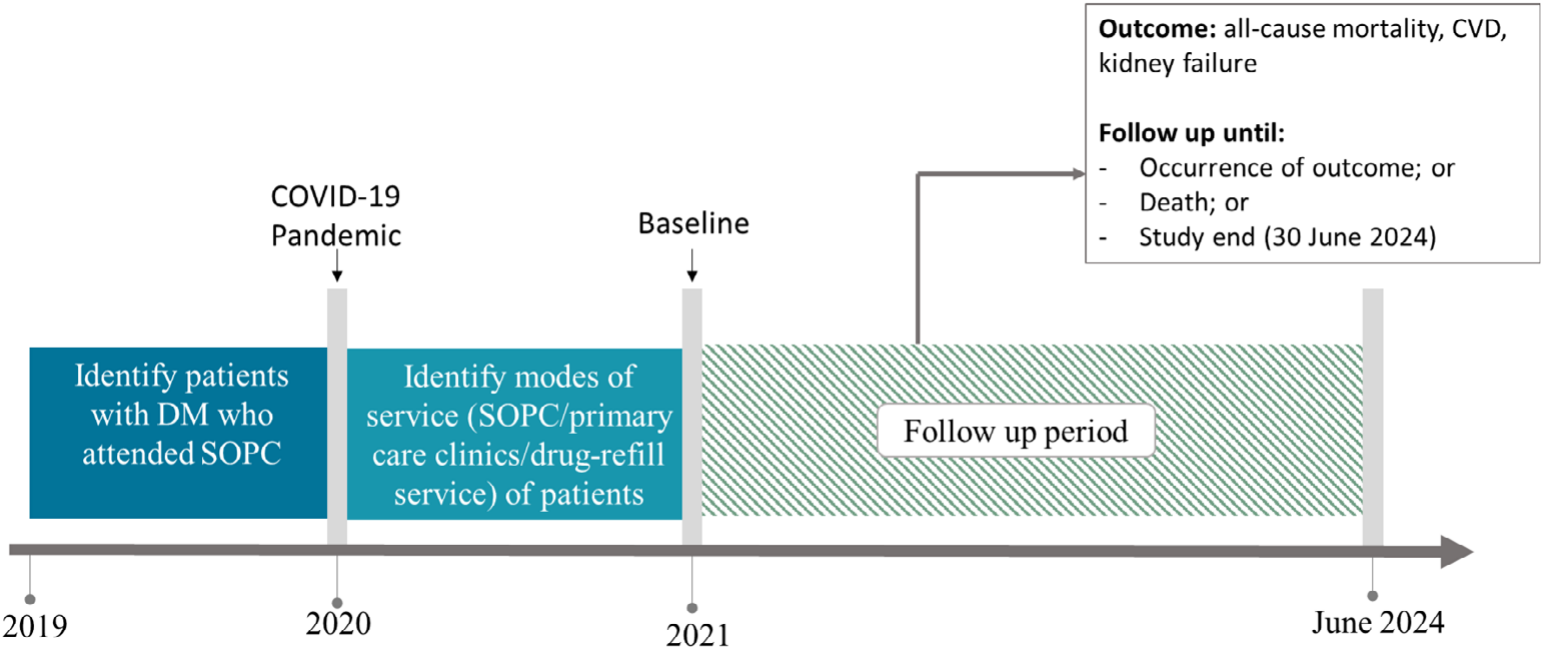
Study design. COVID‐19, coronavirus disease 2019; CVD, cardiovascular diseases; DM, diabetes mellitus; SOPC, specialist outpatient clinic.

We conducted a retrospective cohort study using electronic medical records extracted by the Statistics and Data Science Department of the Hospital Authority (HA) from their Clinical Management System, which provided comprehensive, high‐quality data. The HA is the sole public health care services provider in Hong Kong, managing 43 public hospitals and institutions, 49 SOPCs and 74 general outpatient clinics (GOPCs).^11^ In the 2020–2022 Population Health Survey in Hong Kong, 86% of the patients with DM had medical follow‐up at public clinics or hospitals.^12^ We included patients who were diagnosed with DM from January 2010 to December 2019 and had at least one doctor consultation in an internal medicine SOPC in 2019. Patients with DM were identified based on the International Classification of Primary Care (ICPC) and International Classification of Diseases, Ninth Revision, Clinical Modification (ICD‐9‐CM) (Table S1). Patients were excluded if they were aged <18 years or were diagnosed with CVD or kidney failure on or before December 2020. Patients were observed from 1 January 2021 (baseline) until one of the following: (1) date of death, (2) date of outcome of interest, or (3) the last follow‐up date under the Hospital Authority on or before June 2024. The study design is illustrated in Figure 1.

### 2.2 Definition of exposure and comparator groups

We identified the use of modes of service, including internal medicine SOPC, primary care clinics, and drug‐refill service, for patients with DM from January to December 2020. The internal medicine SOPC in Hong Kong provides specialist consultation services to complicated or severe diabetic patients, such as patients with complications or features of endocrinopathies, those aged <30 years, and pregnant women, who are referred by GOPCs, private practitioners, or family doctors.^5^ SOPC does not include family medicine clinics; family medicine clinics are grouped with GOPC as primary care clinics in Hong Kong. Patients with disrupted SOPC services can have access to family medicine (FM) clinics. There is an overlap between doctors' training and experience in the FM clinics and GOPC, with most being specialists and trainees in family medicine. A small proportion (<10%) of doctors in the GOPC are primary care service doctors without any postgraduate speciality training. There are no non‐general practitioner specialists working in FM clinics or GOPC. The main differences between the FM clinics and GOPC are the drug formularies and access to special investigations, for example, CT scan. Patients with DM are normally managed by the doctor, supported by a multidisciplinary team (MDT) of nurses, pharmacists, optometrists, dieticians, and other health professionals in both SOPC and primary care clinics in Hong Kong. In Hong Kong, public hospitals or clinics have been providing telehealth services to patients during the COVID‐19 pandemic. The SOPC or primary care clinic services delivered through tele‐consultation were also counted as the SOPC or primary care clinic attendance in our analyses. We defined disruption of the internal medicine SOPC service in 2020 as the absence of doctor consultation records in the internal medicine SOPC in 2020. The comparator group was patients who attended the internal medicine SOPC in 2020. For patients with DM affected by SOPC follow‐up disruptions, alternative modes of service for SOPC include primary care clinics and drug‐refill services. Primary care clinic attendance was defined as any doctor consultation records in GOPCs. Drug‐refill service, defined as repeating dispensing medication without clinic attendance, was identified by drug prescription records without doctor consultation in the same month in SOPC or primary care clinics. Primary care clinics and drug‐refill services were not exclusive services.

### 2.3 Outcomes

The primary outcome of this study was all‐cause mortality. Secondary outcomes included the incidence of CVD and kidney failure. CVD was defined as the first occurrence of coronary heart disease (CHD), stroke, or heart failure (HF), which were identified using ICD‐9‐CM or ICPC‐2 codes (Table S1). Kidney failure was defined by ICD‐9‐CM codes or estimated glomerular filtration rate (eGFR) <15 mL/min/1.73 m^2^ (Table S1).

### 2.4 Baseline covariates

The baseline covariates included sex, age, smoking status (yes or no), blood pressure (BP), glycated haemoglobin (HbA1c), lipid profile (low‐density lipoprotein cholesterol [LDL‐C]; high‐density lipoprotein cholesterol [HDL‐C]; total cholesterol; triglyceride), body mass index (BMI), eGFR (calculated by the 2009 Chronic Kidney Disease Epidemiology Collaboration (CKD‐EPI) equation^13^), urine albumin‐to‐creatinine ratio, Charlson Comorbidity Index (CCI), disease duration, comorbidities (yes or no; hypertension), medication use (yes or no; insulin, oral antidiabetic drugs, lipid‐lowering drugs, renin‐angiotensin‐system blockers, beta blockers and other antihypertensive drugs), and healthcare utilisation in 2020, including internal medicine SOPC (frequency), primary care clinics (frequency), drug‐refill services (frequency), accident and emergency (A&E) (yes or no) and hospital admission (yes or no). The smoking status in the datasets classifies people into “smoker”, “non‐smoker” and “unknown”. We assigned people with unknown smoking status to the “non‐smokers” group. All the disease definitions of baseline characteristics are listed in Table S1.

### 2.5 Statistical analysis

Descriptive baseline characteristics were presented as mean ± SD for continuous variables or number (percentage) for categorical variables. As for missing baseline variables, we applied multiple imputations using the “mi impute” command in Stata to address the potential selection bias from incomplete variables. The chained equation method was used to impute five times for each missing value,^14^ and the results were combined based on Rubin rules.^15^

Differences in baseline characteristics between patients with and without visits to internal medicine SOPC, primary care clinics and drug‐refill service in 2020 were assessed using chi‐square tests for categorical variables or independent *t* tests for continuous variables. Incidence rates of all‐cause mortality, CVD, and kidney failure (cases per 1000 person‐month) were calculated. The multivariable‐adjusted Cox proportional hazards models were used to estimate the hazard ratios (HRs) and 95% confidence intervals (95% CIs) for outcome events between patients with and without visits to internal medicine SOPC and alternative modes of care. The assumption of proportional hazards was checked by the plots of the scaled Schoenfeld residuals against time for the covariates. We examined the changes in HbA1c from baseline to 2023 within groups (SOPC attendance and SOPC follow‐up disruption) using the generalized estimating equation model and adjusting for baseline covariates. In addition, subgroup analysis was conducted by age (<65 and ≥65 years), sex (female and male), CCI (<2 and ≥2), disease control (HbA1c <7% and ≥7%), eGFR (<60 and ≥60 mL/min/1.73 m^2^) and smoking status (smoker and non‐smoker). Five sensitivity analyses were conducted: (1) conducting a competing risk Cox regression by using the Fine‐Grey model, with death considered as a competing risk; (2) excluding patients with COVID‐19 to minimise the impact of SARS‐CoV‐2 infection on outcomes of interest (the case definition of COVID‐19 is shown in Table S1); (3) including patients with at least 1 year of follow‐up; (4) including only patients who had at least one SOPC follow‐up from 2021 to 2024; (5) using fine stratification weights to minimise the potential confounding and improve the balance between groups. This method generated strata based on the propensity score before calculating the weights within each stratum. Multivariable logistic regression, adjusting for baseline characteristics, was applied to estimate the propensity score. All statistical tests were two‐sided, and the results were considered significant when the *p*‐value was <0.05. All statistical analysis was performed by Stata version 16.1/MP.

## 3 RESULTS

A total of 96 372 patients with DM who had internal medicine SOPC attendance in 2019 were included in the analysis. The patient inclusion flow is illustrated in Figure S1. Table 1 shows the baseline characteristics of patients with DM. The mean (SD) age of the patients was 67.0 (13.2) years, with 47 839 (49.6%) of them being male. Among them, 82 412 (85.5%) attended doctor consultations in internal medicine SOPC from January to December 2020, and 13 960 (14.5%) did not. Among patients with SOPC attendance in 2020, 51.0%, 52.7%, and 52.1% of the patients achieved HbA1c <7% (American Diabetes Association glycaemic goal) in 2021, 2022, and 2023, respectively. Their median follow‐up interval in 2020 was 4 (IQR: 4–6) months, with an average of 2.76 (SD: 1.58) SOPC visits. Among those who had disruption of SOPC follow‐up in 2020, 10 299 (73.8%) patients had primary care clinic doctor consultation with a mean (SD) frequency of 4.0 (2.2) (Table S2). Among 13 960 patients who were affected by SOPC follow‐up disruption in 2020, 5331 (38.2%) patients had at least one SOPC follow‐up in 2021–2024. Of the remaining 8629 patients without any SOPC follow‐up in 2021–2024, 6629 (76.8%) patients had at least one doctor consultation in primary care clinics during 2021–2024. The baseline characteristics of patients with DM who had disruption of SOPC visit and used the drug‐refill service, or did not, are shown in Table S3. The data completion rates for baseline covariates are shown in Tables S4 and S5.

**TABLE 1:** Baseline characteristics of patients with DM (*N* = 96 372).

Characteristics	Overall	SOPC attendance (*N* = 82 412)	SOPC disruption (*N* = 13 960)	*p* value
Age (years)	66.95 ± 13.20	66.79 ± 13.23	67.86 ± 13.00	<0.001
65 years	54 026 (56.06)	45 922 (55.72)	8104 (58.05)	<0.001
Sex, male	47 839 (49.64)	41 240 (50.04)	6599 (47.27)	<0.001
CCI	1.86 ± 1.43	1.91 ± 1.45	1.58 ± 1.23	<0.001
≥2	37 434 (38.84)	33 330 (40.44)	4104 (29.40)	<0.001
Disease duration (years)	7.35 ± 3.35	7.45 ± 3.30	6.78 ± 3.60	<0.001
Smoker	32 578 (33.80)	28 059 (34.05)	4519 (32.37)	<0.001
A&E attendance in 2020, yes	21 655 (22.47)	18 987 (23.04)	2668 (19.11)	<0.001
Hospital admission in 2020, yes	14 664 (15.22)	13 069 (15.86)	1595 (11.43)	<0.001
Drug‐refill service in 2020, yes	9218 (9.57)	8711 (10.57)	507 (3.63)	<0.001
Primary care clinic attendance in 2020, yes	42 452 (44.05)	32 153 (39.01)	10 299 (73.78)	<0.001
Number of SOPC attendance in 2020	2.36 ± 1.75	2.76 ± 1.58	0.00 ± 0.00	<0.001
Number of drug‐refill service in 2020	0.13 ± 0.45	0.14 ± 0.47	0.06 ± 0.36	<0.001
Number of primary care clinic attendance in 2020	1.65 ± 2.71	1.43 ± 2.67	2.92 ± 2.58	<0.001
Clinical parameters				
HbA1c (%)	7.16 ± 1.30	7.19 ± 1.31	6.96 ± 1.25	<0.001
SBP (mmHg)	137.47 ± 17.97	138.05 ± 18.17	134.00 ± 16.29	<0.001
DBP (mmHg)	75.19 ± 11.32	75.51 ± 11.33	73.31 ± 11.10	<0.001
eGFR (mL/min/1.73 m^2^)	76.10 ± 23.16	75.68 ± 23.47	78.59 ± 21.01	<0.001
UACR (mg/mmol)	12.73 ± 21.67	13.32 ± 22.16	9.25 ± 18.18	<0.001
BMI (kg/m^2^)	25.90 ± 4.59	25.93 ± 4.61	25.71 ± 4.48	<0.001
Lipid panel				
LDL‐C (mmol/L)	2.12 ± 0.72	2.12 ± 0.73	2.15 ± 0.69	<0.001
HDL‐C (mmol/L)	1.30 ± 0.37	1.30 ± 0.37	1.30 ± 0.35	0.11
Total cholesterol (mmol/L)	4.12 ± 0.88	4.11 ± 0.88	4.14 ± 0.84	<0.001
Triglyceride (mmol/L)	1.56 ± 1.09	1.56 ± 1.11	1.53 ± 1.00	0.76
Medicine use				
Insulin	25 517 (26.48)	24 288 (29.47)	1229 (8.80)	<0.001
Oral antidiabetic drugs	57 608 (59.78)	48 859 (59.29)	8749 (62.67)	<0.001
Lipid lowering drugs	66 413 (68.91)	57 741 (70.06)	8672 (62.12)	<0.001
RAS blockers	54 029 (56.06)	47 421 (57.54)	6608 (47.34)	<0.001
Beta blockers	25 129 (26.08)	22 269 (27.02)	2860 (20.49)	<0.001
Other antihypertensive drugs	54 202 (56.24)	46 672 (56.63)	7530 (53.94)	<0.001
Comorbidities				
Hypertension	64 814 (67.25)	53 939 (65.45)	10 875 (77.90)	<0.001

*Note*: Data are presented in mean ± SD or number (percentage) as appropriate.

Abbreviations: A&E, accident and emergency; BMI, body mass index; CCI, Charlson Comorbidity Index; DBP, diastolic blood pressure; DM, diabetes mellitus; eGFR, estimated glomerular filtration rate; HbA1c, glycated haemoglobin; HDL‐C, high‐density lipoprotein cholesterol; LDL‐C, low‐density lipoprotein cholesterol; RAS, renin‐angiotensin‐system; SBP, systolic blood pressure; SD, standard deviation; SOPC, specialist outpatient clinic; UACR, urinary albumin‐to‐creatinine ratio.

Table 2 shows the association between SOPC follow‐up disruption and all‐cause mortality, the incidence of CVD, and kidney failure. Over a follow‐up of 3 851 768 person‐months, the total number of death events was 9848. Compared with those who continued SOPC follow‐up in 2020, patients who did not visit SOPC had a 20% (HR 1.20, 95% CI 1.12–1.29) higher risk of all‐cause mortality, a 12% (HR 1.12, 95% CI 1.05–1.20) higher risk of overall CVD incidence, an 18% (HR 1.18, 95% CI 1.04–1.35) higher risk of HF incidence and an 11% (HR 1.11, 95% CI 1.00–1.24) higher risk of kidney failure incidence. There was no significant association between disruption of SOPC follow‐up and kidney failure incidence.

**TABLE 2:** Association between disruption of SOPC visit and all‐cause mortality, CVD and kidney failure incidence among patients with DM (*N* = 96 372).

Outcomes	SOPC disruption (*N* = 13 960)			SOPC attendance (Reference) (*N* = 82 412)			Adjusted HR (95% CI)
	Event	Person‐month	Crude incidence rate (cases, 1000 person‐month)	Event	Person‐month	Crude incidence rate (cases, 1000 person‐month)	
All‐cause mortality	1294	559 305	2.31 (2.19, 2.44)	8554	3 292 463	2.60 (2.54, 2.65)	1.20 (1.12, 1.29)***
CVD	1476	554 778	2.66 (2.53, 2.80)	8469	3 281 778	2.58 (2.53, 2.64)	1.12 (1.05, 1.20)**
CHD	714	571 740	1.25 (1.16, 1.34)	4022	3 377 353	1.19 (1.15, 1.23)	1.07 (0.97, 1.19)
HF	327	579 779	0.56 (0.51, 0.63)	2442	3 411 215	0.72 (0.69, 0.74)	1.18 (1.04, 1.35)*
Stroke	598	573 195	1.04 (0.96, 1.13)	3171	3 394 897	0.93 (0.90, 0.97)	1.08 (0.97, 1.20)
Kidney failure	535	575 832	0.93 (0.85, 1.01)	5217	3 351 680	1.56 (1.51, 1.60)	1.11 (1.00, 1.24)*

*Note*: Adjusted for age, sex, Charlson Comorbidity Index, smoking, disease duration, systolic blood pressure, diastolic blood pressure, hospital admission (yes or no), accident and emergency (yes or no), primary care clinic (yes or no), drug‐refill service (yes or no), frequency of SOPC visits in 2020, frequency of primary care clinic visits in 2020, frequency of drug‐refill service in 2020, glycated haemoglobin, estimated glomerular filtration rate, urinary albumin‐to‐creatinine ratio, body mass index, low‐density lipoprotein cholesterol, high‐density lipoprotein cholesterol, total cholesterol, triglycerides, insulin, oral antihyperglycaemic drugs, lipid lowering drugs, renin‐angiotensin‐system blockers, beta blockers, other antihypertensive drugs and hypertension.

Abbreviations: CHD, coronary heart disease; CI, confidence interval; CVD, cardiovascular disease; DM, diabetes mellitus; HF, heart failure; HR, hazard ratio; SOPC, specialist outpatient clinics.

* *p* < 0.05;

** *p* < 0.01;

*** *p* < 0.001.

Table 3 shows the association between alternative modes of care and all‐cause mortality, incidence of CVD, and kidney failure among those who had disruption of SOPC in 2020. Primary care clinic doctor consultations were associated with a 28% (HR 0.72, 95% CI 0.60–0.87), 16% (HR 0.84, 95% CI 0.72–0.99), and 35% (HR 0.65, 95% CI 0.46–0.93) lower risk of all‐cause mortality, CVD, and HF incidence, respectively, compared with those without any primary care clinic doctor consultations. We found no association between drug‐refill service and the risk of mortality, CVD, or kidney failure.

**TABLE 3:** Association between alternative modes of care and all‐cause mortality, CVD, and kidney failure incidence among patients with DM who had disruption of SOPC visit (*N* = 13 960).

Outcomes	Alternative modes of care			Reference			Adjusted HR (95% CI)
	Event	Person‐month	Crude incidence rate (cases, 1000 person‐month)	Event	Person‐month	Crude incidence rate (cases, 1000 person‐month)	
**Primary care clinic**							
All‐cause mortality	815	416 464	1.96 (1.83, 2.10)	479	142 841	3.35 (3.07, 3.67)	0.72 (0.60, 0.87)***
CVD	1125	408 448	2.75 (2.60, 2.92)	351	146 330	2.40 (2.16, 2.66)	0.84 (0.72, 0.99)*
CHD	554	421 064	1.32 (1.21, 1.43)	160	150 676	1.06 (0.91, 1.24)	0.87 (0.68, 1.10)
HF	228	427 994	0.53 (0.47, 0.61)	99	151 785	0.65 (0.54, 0.79)	0.65 (0.46, 0.93)*
Stroke	450	422 751	1.06 (0.97, 1.17)	148	150 444	0.98 (0.84, 1.16)	0.94 (0.72, 1.21)
Kidney failure	350	425 959	0.82 (0.74, 0.91)	185	149 873	1.23 (1.07, 1.43)	0.83 (0.62, 1.09)
**Drug‐refill**							
All‐cause mortality	85	19 516	4.36 (3.52, 5.39)	1209	539 789	2.24 (2.12, 2.37)	0.86 (0.59, 1.26)
CVD	51	20 180	2.53 (1.92, 3.33)	1425	534 598	2.67 (2.53, 2.81)	0.81 (0.50, 1.31)
CHD	22	20 859	1.05 (0.69, 1.60)	692	550 881	1.26 (1.17, 1.35)	1.28 (0.50, 3.23)
HF	14	20 986	0.67 (0.40, 1.13)	313	558 793	0.56 (0.50, 0.63)	0.78 (0.30, 2.09)
Stroke	22	20 762	1.06 (0.70, 1.61)	576	552 433	1.04 (0.96, 1.13)	0.64 (0.33, 1.25)
Kidney failure	28	20 781	1.35 (0.93, 1.95)	507	555 051	0.91 (0.84, 1.00)	1.19 (0.53, 2.66)

*Note*: Adjusted for age, sex, Charlson Comorbidity Index, smoking, disease duration, systolic blood pressure, diastolic blood pressure, hospital admission (yes or no), accident and emergency (yes or no), primary care clinic (yes or no), drug‐refill service (yes or no), frequency of primary care clinic visits in 2020, frequency of drug‐refill service in 2020, glycated haemoglobin, estimated glomerular filtration rate, urinary albumin‐to‐creatinine ratio, body mass index, low‐density lipoprotein cholesterol, high‐density lipoprotein cholesterol, total cholesterol, triglycerides, insulin, oral antihyperglycaemic drugs, lipid lowering drugs, renin‐angiotensin‐system blockers, beta blockers, other antihypertensive drugs and hypertension.

Abbreviations: CHD, coronary heart disease; CI, confidence interval; CVD, cardiovascular disease; DM, diabetes mellitus; HF, heart failure; HR, hazard ratio; SOPC, specialist outpatient clinics.

* *p* < 0.05;

*** *p* < 0.001.

The HbA1c changes from baseline to 2023 were not significant in either patients with SOPC attendance in 2020 (mean difference [95% CI]: −0.004% [−0.013%, 0.005%], *p* = 0.366) or those with SOPC follow‐up disruption in 2020 (mean difference [95% CI]: 0.014% [−0.009%, 0.036%], *p* = 0.229). The difference in HbA1c changes between groups was not significant (difference in difference [95% CI]: 0.018% [−0.006%, 0.042%], *p* = 0.145).

Subgroup analyses were conducted on the outcomes of all‐cause mortality, overall CVD, and kidney failure, as shown in Figure S2. The results were largely consistent with the main analysis for different subgroups. Among these subgroups, patients with a CCI of 2 or over were more likely to die if they had SOPC disruption, compared with their opposing subgroups. Among those who had SOPC disruption, patients who were female and aged 65 or over received more benefit from primary care clinics than their opposing subgroups. In addition, the results from sensitivity analyses were similar to the main analysis, as shown in Tables S6–S15.

## 4 DISCUSSION

In this population‐based cohort study of SOPC patients with DM, we found that SOPC follow‐up disruption was associated with a greater risk of all‐cause mortality and CVD incidence. In patients who did not visit SOPC in 2020, patients who had primary care clinic doctor consultations showed a lower risk of all‐cause mortality and HF than those who did not, while drug‐refill service was not associated with risk reductions of complications and mortality. The results inform healthcare policy that primary care clinics have a beneficial effect on patients with SOPC disruption and can serve as a beneficial alternative mode of care in future public health crises.

We found that patients with DM who did not have doctor consultations in SOPC had an increased risk of CVD and all‐cause mortality. Higher rates of CVD and mortality among patients with DM were reported during the COVID‐19 pandemic, but these studies did not examine the contribution of disrupted outpatient services to the increased rates.^16^ This study adds to previous findings about the relative risk of SOPC disruption associated with CVD and mortality. Patients under care in SOPC are more likely to have diabetic complications, multiple comorbidities, and other complicated medical needs. In a DM care report from the Hospital Authority in Hong Kong, more patients with regular follow‐ups in SOPC had chronic kidney disease and acute hospitalisation due to hyperglycaemia compared with those in primary care.^6^ For patients who did not have follow‐ups in SOPC, the lack of monitoring of complications and review of treatment might increase the risk of the progression of complications and even death. Although some studies found no significant difference in patients' disease control between before and during the COVID‐19 pandemic,^17^ most studies showed poorer glycaemic, blood pressure, and lipid control among patients with DM during the COVID‐19 pandemic when outpatient visits were largely reduced.^18^, ^19^ We did not observe a significant difference in the HbA1c changes from baseline to follow‐up between patients with and without SOPC attendance in 2020, which indicated that SOPC visits in 2020 were not associated with better HbA1c in the follow‐up period. Therefore, the reduction in cardiorenal events in patients with SOPC attendance was not reflected by changes in blood glucose. The underlying mechanism of the association between SOPC service disruption and increased risk of outcomes is complex. It includes not only the effect of blood glucose control but also an interaction with the effect of the control of other clinical parameters, such as blood pressure, blood lipid, and estimated glomerular filtration rate (eGFR), lifestyle, and self‐care. Further studies are needed to investigate the significant factors and their interactions that contribute to the increase in cardiorenal events. As for the subgroup analysis, among patients with CCI <2, SOPC follow‐up disruption was not associated with a higher risk of all‐cause mortality. As patients with CCI <2 had fewer comorbidities and lower all‐cause mortality risk, the effect of SOPC follow‐up disruption in 2020 had little effect on the mortality rates of these patients.

Primary care clinic doctor consultations served as an alternative mode of care in diabetic patients who had disrupted SOPC follow‐up, with a 30% relative risk reduction of HF and all‐cause mortality. In primary care clinics, patients with DM receive regular assessments of cardiovascular risk factors, such as blood glucose, blood pressure, and blood lipids. In those who have suboptimal control of risk factors, primary care practitioners can provide timely medication adjustments and monitor the adverse effects of the medication. Furthermore, education provided by primary care practitioners to equip patients with knowledge and skills in diabetic self‐care, including maintaining a healthy lifestyle, self‐monitoring, and adherence to medication, is crucial to disease control. For patients who did not have follow‐ups in SOPC, these services provided by primary care clinics can still help them stabilise blood glucose, blood pressure, and blood lipids. Stabilised and good control of these risk factors can reduce the risk of occurrence or slow the progression of cardiovascular complications, and therefore reduce the risk of premature deaths.^20^, ^21^ Additionally, patients with DM in primary care clinics undergo regular screening of diabetes‐related complications, such as kidney diseases, diabetic retinopathy, and diabetic foot.^5^ Regular screenings enable early detection and treatment of these complications to reduce the risk of progression and related deaths. In the subgroup analyses, among patients aged less than 65, patients who had primary care clinic attendance exhibited a higher risk of kidney failure. The low incidence rate of kidney failure in younger patients (0.37 per 1000 person‐month in age <65 years versus 1.4 per 1000 person‐month in age ≥65 years) might limit the estimation of the hazard ratio due to limited statistical power. Further research is needed to explore the effect of primary care clinic attendance on kidney failure among younger patients.

Drug‐refill service has been widely practised to reduce the rising physician consultation burden due to the increasing prevalence of DM.^22^, ^23^ A prior study suggested a greater proportion of patients with drug‐refill service achieved glycaemic control targets during half a year of follow‐up than those with non‐refill.^10^ However, the effectiveness of drug‐refill service can be affected by many factors, such as patient compliance and medication adherence. In our study, drug‐refill service showed no association with risk reduction of CVD, kidney failure, and all‐cause mortality among patients with DM who had SOPC disruption. This might be due to the lack of drug or dose adjustments or low medication adherence. In addition, the small sample size of the drug‐refill service group may result in lower statistical power to detect the difference. A future study with a large sample size is needed to evaluate the effect of drug‐refill service on the risk reduction of CVD, kidney failure, and mortality in patients with DM.

To our knowledge, this is the first study to evaluate the association of alternative modes of care with the risk of all‐cause mortality, CVD, and kidney failure in SOPC patients with DM who did not have a doctor consultation in SOPC during the COVID‐19 waves. Using a large sample size of over 90 000 patients with DM, this study was territory‐wide representative of the patients with DM in Hong Kong. The results of this study have important implications for the management of patients with DM during future public health crises. The findings can inform healthcare policy that primary care clinics should be provided as an alternative mode of service for patients who have had a disruption of SOPC follow‐up. This will help policymakers optimise healthcare resource allocation and reorganisation of services during an acute public health crisis, to ensure the quality of care for patients with DM can be maintained. This study also has some limitations. First, the follow‐up duration of this study was relatively short and the findings might not reflect the long‐term impact. Second, the analysis was restricted to the healthcare in Hong Kong, which might influence the generalisability to other countries or territories with different healthcare systems. Third, the analysis used the data from public hospitals only, without involving the data from private sectors. Given the overwhelmed public healthcare in Hong Kong during the COVID‐19 pandemic, patients who did not visit outpatient clinics in public sectors may seek private medical care. The lack of including the data from private hospitals could have introduced bias, but the effect was likely to be low since only 6.0% of patients with diabetes visited private clinics or hospitals during the COVID‐19 pandemic, according to the Population Health Survey conducted between November 2020 and January 2022 in Hong Kong.^12^ We did not have access to the data on the clinics or centres operated by the Department of Health (DH). The DH operates clinics providing maternal and child health, student health services, dental services, etc., but does not provide specific services for diabetes care. Although the omission of data from the Department of Health could have introduced bias to our results, the possibility was low given their different service scope and patient populations. Fourth, physicians in the SOPC may lengthen the follow‐up interval for the low‐risk group due to patients' requests and/or social distancing policy. While we have adjusted the disease severity (e.g., disease duration, HbA1c, blood pressure) at baseline and frequency of SOPC visits, primary care clinics visits and drug‐refill service in 2020 in the Cox regression model to minimise confounding by indication and the duration of follow‐up, the risk of residual confounding might exist. Fifth, patients who had SOPC follow‐up disruption might be more likely to default follow‐up visits because of lower health awareness and other unmeasured social factors, which could be a potential confounder in this study. As these social factors were not captured in this study, their effects on the outcomes were uncertain. Future studies considering these social factors should be conducted. Sixth, while we have adjusted for observable confounders (e.g., disease duration, HbA1c, blood pressure) to reduce the bias and conducted a sensitivity analysis using fine stratification weights to improve the balance between groups, we cannot rule out the possibility of residual confounders due to unmeasured factors and the inherent limitations of observational studies.

## 5 CONCLUSION

SOPC follow‐up disruption was associated with an increased risk of mortality and CVD in patients with DM. Primary care clinics were a beneficial mode of care in mitigating these risks in patients experiencing SOPC follow‐up disruption. These findings inform public policy that primary care clinics can serve as alternative modes of care to optimise healthcare resource allocation in future public health crises.

## AUTHOR CONTRIBUTIONS

Study design and conception: Y.K.Y., E.Y.F.W.; data acquisition: Y.K.Y., E.Y.F.W.; statistical analysis: M.L.; data interpretation: Y.K.Y., C.L.K.L., E.Y.F.W.; manuscript draft: Y.K.Y.; critical revision: Y.K.Y., J.Q., K.A.G., G.K.K.L., I.L.M., C.S.L., I.C.K.W., D.V.K.C., W.W.K.K., C.L.K.L., E.Y.F.W.; funding acquisition: C.L.K.L., E.Y.F.W.; supervision or mentorship: C.L.K.L., E.Y.F.W. All authors read and approved the manuscript for submission.

## FUNDING INFORMATION

The funder had no role in the design and conduct of the study; collection, management, analysis, and interpretation of the data; and preparation of the manuscript. This work was funded by the Health and Medical Research Fund, Health Bureau, Government of Hong Kong Special Administrative Region (grant no: COVID19F08).

## CONFLICT OF INTEREST STATEMENT

EYFW has received research grants from the Health Bureau, the Hong Kong Research Grants Council, Narcotics Division, Security Bureau, Social Welfare Department, Labour and Welfare Bureau of the Government of the Hong Kong SAR, and the National Natural Science Foundation of China; serves as a member of the Core Team for the Expert Group on Drug Registration of the Pharmacy and Poisons Board, and is the director of Advance Data Analytics for Medical Science (ADAMS) Limited (HK). These are outside the submitted work. ICKW received research grants from Amgen, Janssen, GSK, Novartis, Pfizer, Bayer, Bristol‐Myers Squibb, and Takeda, the Institute for Health Research in England, the European Commission, the National Health and Medical Research Council in Australia, the European Union's Seventh Framework Programme for research, technological development, the Research Grants Council Hong Kong, and the Health and Medical Research Fund Hong Kong; consulting fees from IQVIA and the World Health Organization; payment for expert testimony for the Appeal Court in Hong Kong; serves on advisory committees for the Member of the Pharmacy and Poisons Board; is a member of the Expert Committee on Clinical Events Assessment Following COVID‐19 Immunization; is a member of the Advisory Panel on COVID‐19 Vaccines of the Hong Kong Government; is the non‐executive director of Jacobson Pharma Corp. Ltd. in Hong Kong; and is the founder and director of Therakind Limited (UK), Advance Data Analytics for Medical Science (ADAMS) Limited (HK) and OCUS Innovation Limited (HK, Ireland and UK).

## PEER REVIEW

The peer review history for this article is available at https://www.webofscience.com/api/gateway/wos/peer‐review/10.1111/dom.70069.

## ETHICS STATEMENT

This study was approved by the Severance Hospital Institutional Review Board (IRB No. 4‐2022‐0248) and the Institutional Review Board of the University of Hong Kong Hospital Authority Hong Kong West Cluster (ref.: UW 21‐297).

## Supporting information


**Data S1.** Figures.

**Data S2.** Tables.

## Data Availability

Data will not be made available to others because the data custodians have not given permission.
