# Supplementary material for: Disrupted specialist outpatient services and alternative modes of service for patients with diabetes mellitus: A population‐based, retrospective cohort study in Hong Kong
Source: Diabetes Obes Metab. 2025 Aug 28;27(11):6615–23. doi: 10.1111/dom.70069 (PMC12515776; doi:10.1111/dom.70069)
Supplement: Supplementary file 1 — Data S1. Figures. [file DOM-27-6615-s002.docx]

SFigure 1 Flowchart of patient with DM

DM: diabetes mellitus; SOPC: specialist outpatient clinic; CVD: cardiovascular disease


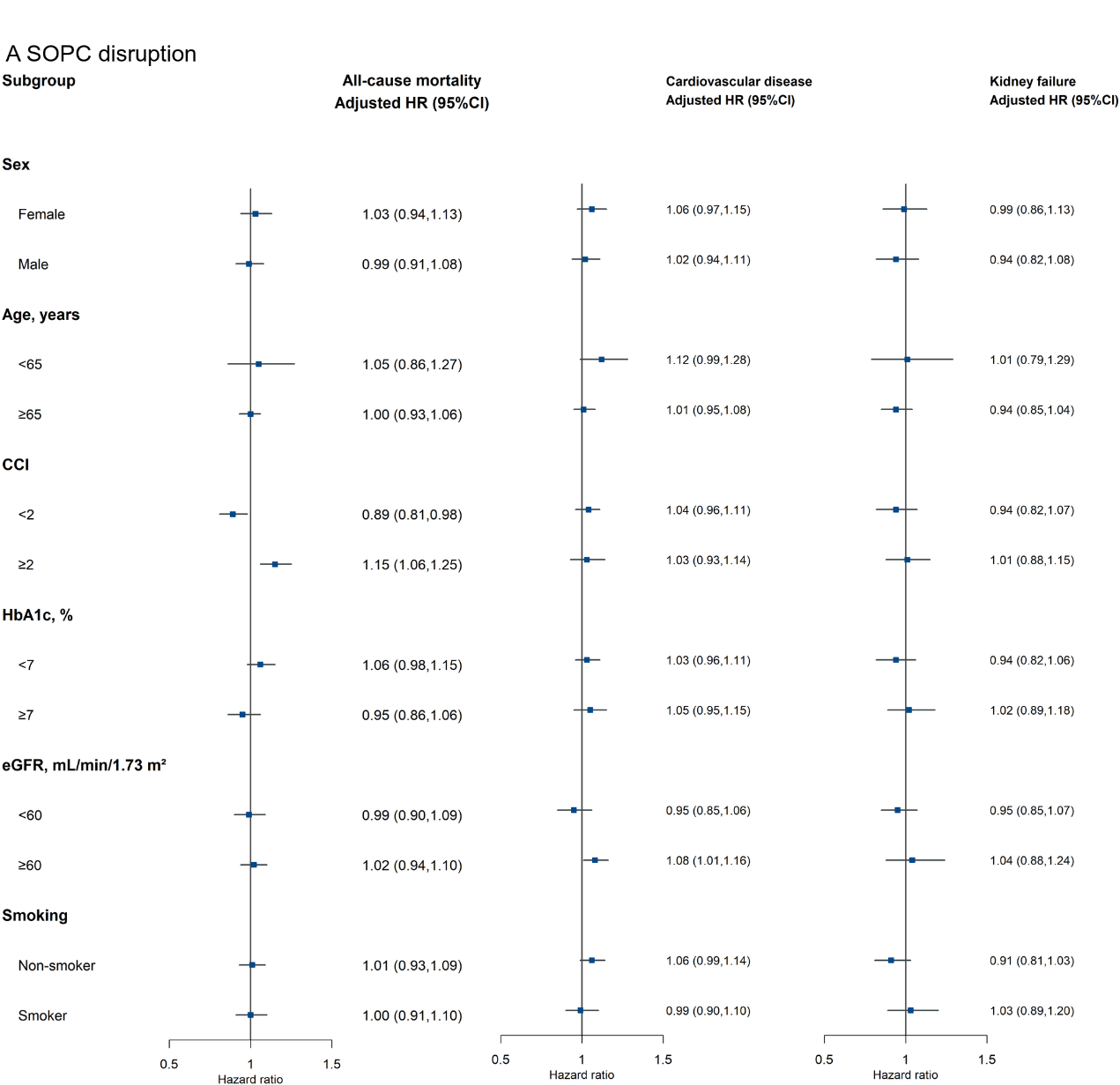


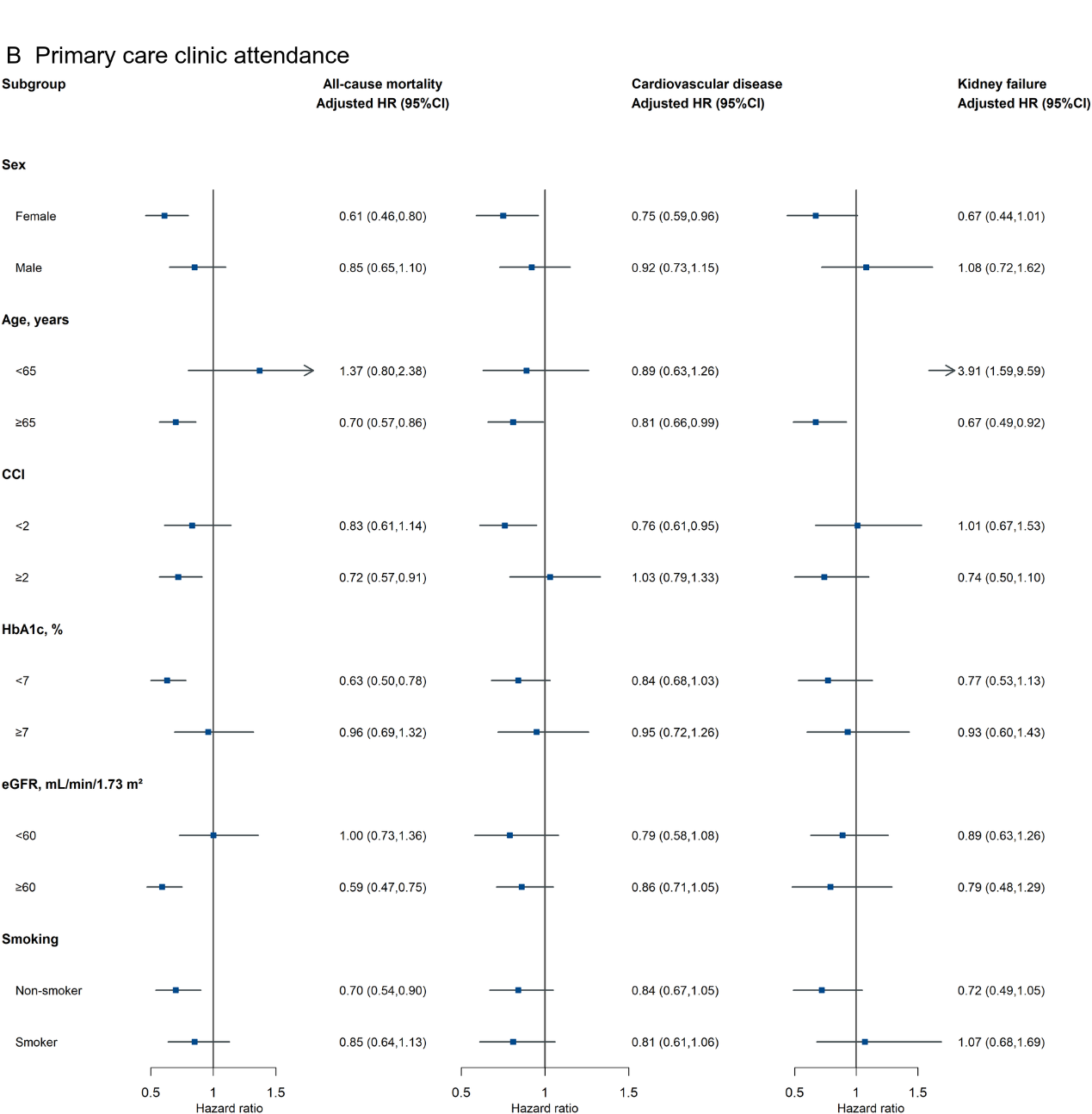


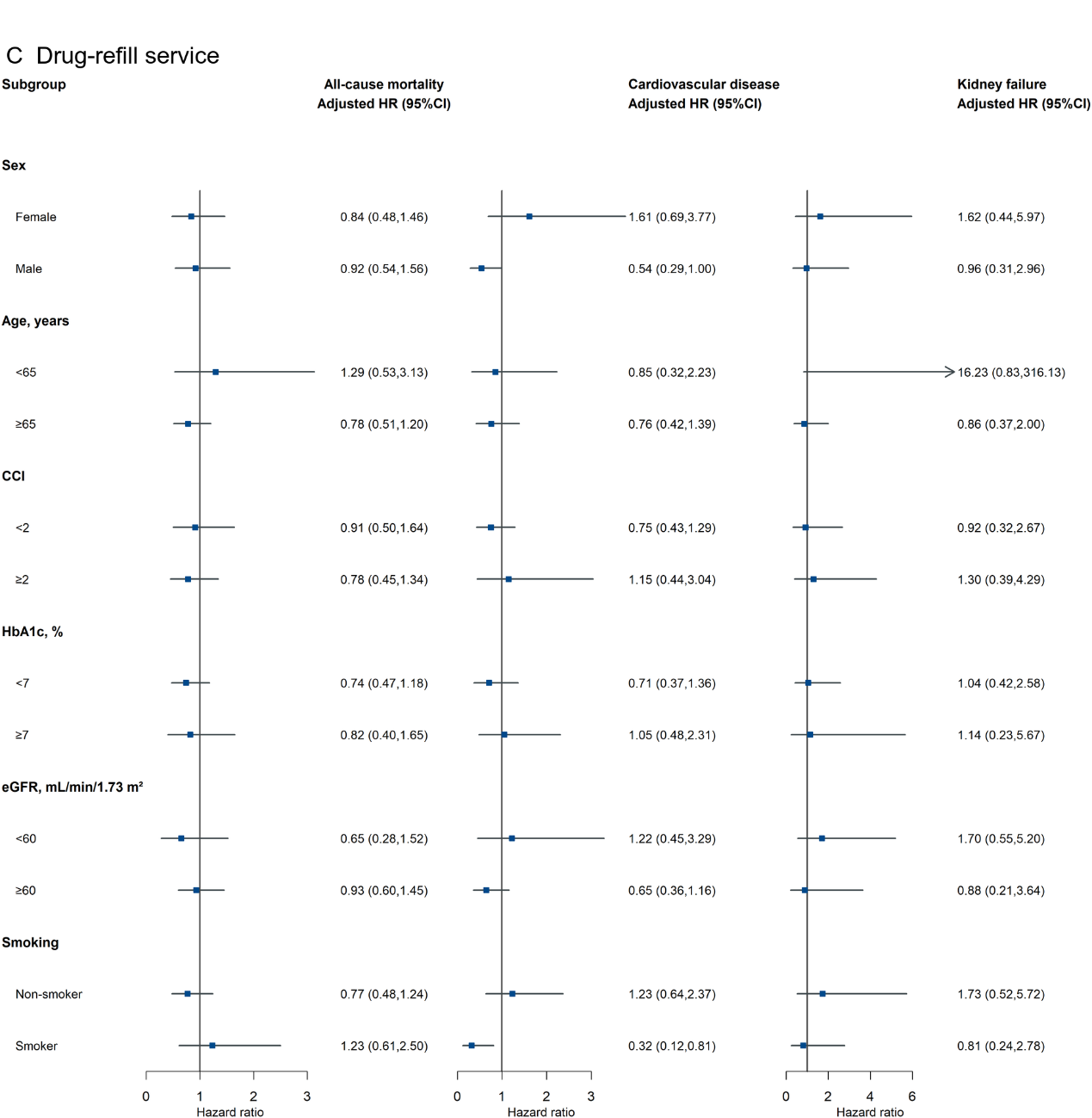


**Figure S2 Adjusted hazard ratios (HRs) of patients with DM in subgroups by multivariable Cox proportional hazards regressions**

A: patients with disruption of SOPC visits compared with patients with SOPC attendance; B: patients had primary care clinic attendance compared with patients did not have primary care clinic attendance; C: patients had drug refill compared with patients did not drug refill;

Footnotes: SOPC: specialist outpatient clinic; CCI: Charlson Comorbidity Index; HbA1c: glycated hemoglobin; eGFR: estimated glomerular filtration rate; HR: hazard ratio; CI: confidence interval;

HRs were adjusted for age, sex, Charlson Comorbidity Index, smoking, disease duration systolic blood pressure, diastolic blood pressure, hospital admission (yes or no), accident and emergency (yes or no), primary care clinic (yes or no), drug-refill service (yes or no), frequency of SOPC visits in 2020 (only SOPC disruption adjusted), frequency of primary care clinic visits in 2020, frequency of drug-refill service in 2020, glycated hemoglobin, estimated glomerular filtration rate, urinary albumin-to-creatinine ratio, body mass index, low-density lipoprotein cholesterol, high-density lipoprotein cholesterol, total cholesterol, triglycerides, insulin, oral antihyperglycemic drugs, lipid lowering drugs, renin-angiotensin-system blockers, beta blockers, other antihypertensive drugs and hypertension.
