# Supplementary material for: Disrupted specialist outpatient services and alternative modes of service for patients with diabetes mellitus: A population‐based, retrospective cohort study in Hong Kong
Source: Diabetes Obes Metab. 2025 Aug 28;27(11):6615–23. doi: 10.1111/dom.70069 (PMC12515776; doi:10.1111/dom.70069)
Supplement: Supplementary file 2 — Data S2. Tables. [file DOM-27-6615-s001.docx]

**Table S1 Definition of the diseases**

| **Disease** | | **ICPC-2** | **ICD-9-CM** | **Others** |
| --- | --- | --- | --- | --- |
| **DM** | | T89-T90 | 250 | NA |
| **Kidney failure** | | NA | 585.5, 585.6, 586, V42.0, V56, 39.95, 54.98, 55.6 | eGFR <15 mL/min/1.73 m^2^ |
| **CVD** | **CHD** | K74-K76 | 410-414, 429.79 | NA |
|  | **Stroke** | K89-K91 | 430-438 | NA |
|  | **HF** | K77 | 428, 402.01, 402.11, 402.91 | NA |
| **COVID-19** | | A77 | NA | Positive results from PCR testing, or administration of antiviral treatments of Paxlovid or Molnupiravir |

Notes: ICPC-2, the International Classification of Primary Care-2; ICD-9-CM, the International Classification of Diseases, Ninth Edition, Clinical Modification; DM: diabetes mellitus; CVD, cardiovascular disease; CHD: coronary heart disease; HF: heart failure; eGFR, estimated glomerular filtration rate; NA, not applicable; PCR: polymerase chain reaction.

**Table S2 Baseline characteristics of patients with DM who had disruption of SOPC visit by primary care clinic attendance (N=** **13,960)**

| Characteristics | Overall | Primary care clinic attendance (no, N=3,661) | Primary care clinic attendance (yes, N=10,299) | P value |
| --- | --- | --- | --- | --- |
| Age (years) | 67.86±13.00 | 66.30±15.67 | 68.42±11.86 | <0.001 |
| > 65 years | 8,104 (58.05) | 1,939 (52.96) | 6,165 (59.86) | <0.001 |
| Sex, male | 6,599 (47.27) | 1,856 (50.70) | 4,743 (46.05) | <0.001 |
| CCI | 1.58±1.24 | 1.71±1.33 | 1.53±1.20 | <0.001 |
| ≥ 2 | 4,104 (29.40) | 1,263 (34.50) | 2,841 (27.59) | <0.001 |
| Disease duration (years) | 6.78±3.60 | 6.67±3.55 | 6.82±3.62 | 0.03 |
| Smoker | 4,519 (32.37) | 1,251 (34.17) | 3,268 (31.73) | 0.007 |
| A&E attendance in 2020, yes | 2,668 (19.11) | 589 (16.09) | 2,079 (20.19) | <0.001 |
| Hospital admission in 2020, yes | 1,595 (11.43) | 426 (11.64) | 1,169 (11.35) | 0.64 |
| Drug-refill service in 2020, yes | 507 (3.63) | 247 (6.75) | 260 (2.52) | <0.001 |
| Number of drug-refill service in 2020 | 0.06±0.36 | 0.11±0.51 | 0.04±0.29 | <0.001 |
| Number of primary care clinic attendance in 2020 | 2.92±2.58 | 0.00±0.00 | 3.96±2.22 | <0.001 |
| Clinical parameters |  |  |  |  |
| HbA1c (%) | 6.96±1.25 | 7.34±1.68 | 6.83±1.02 | <0.001 |
| SBP (mmHg) | 134.00±16.29 | 137.41±18.85 | 132.79±15.10 | <0.001 |
| DBP (mmHg) | 73.31±11.10 | 76.10±11.94 | 72.32±10.61 | <0.001 |
| eGFR (mL/min/1.73 m^2^) | 78.59±21.02 | 78.95±24.00 | 78.46±19.84 | 0.227 |
| UACR (mg/mmol) | 9.25±18.18 | 12.10±20.28 | 8.23±17.26 | <0.001 |
| BMI (kg/m^2^) | 25.71±4.48 | 25.74±4.51 | 25.70±4.47 | 0.6132 |
| Lipid panel |  |  |  |  |
| LDL-C (mmol/L) | 2.15±0.69 | 2.27±0.80 | 2.11±0.64 | <0.001 |
| HDL-C (mmol/L) | 1.30±0.35 | 1.28±0.36 | 1.31±0.35 | <0.001 |
| Total cholesterol (mmol/L) | 4.14±0.84 | 4.27±0.97 | 4.09±0.78 | <0.001 |
| Triglyceride (mmol/L) | 1.53±1.00 | 1.62±1.21 | 1.49±0.91 | <0.001 |
| Medicine use |  |  |  |  |
| Insulin | 1,229 (8.80) | 526 (14.37) | 703 (6.83) | <0.001 |
| Oral antidiabetic drugs | 8,749 (62.67) | 956 (26.11) | 7,793 (75.67) | <0.001 |
| Lipid lowering drugs | 8,672 (62.12) | 1,180 (32.23) | 7,492 (72.74) | <0.001 |
| RAS blockers | 6,608 (47.34) | 1,014 (27.70) | 5,594 (54.32) | <0.001 |
| Beta blockers | 2,860 (20.49) | 422 (11.53) | 2,438 (23.67) | <0.001 |
| Other antihypertensive drugs | 7,530 (53.94) | 1,104 (30.16) | 6,426 (62.39) | <0.001 |
| Comorbidities |  |  |  |  |
| Hypertension | 10,875 (77.90) | 2,323 (63.45) | 8,552 (83.04) | <0.001 |

Data are presented in mean ± SD or number (percentage) as appropriate.

DM: diabetes mellitus; CCI: Charlson Comorbidity Index; A&E: accident and emergency; SOPC: specialist outpatient clinic; HbA1c: glycated hemoglobin; SBP: systolic blood pressure; DBP: diastolic blood pressure; eGFR: estimated glomerular filtration rate; BMI: body mass index; LDL-C: low-density lipoprotein cholesterol; HDL-C: high-density lipoprotein cholesterol; UACR: urinary albumin-to-creatinine ratio; RAS: renin-angiotensin-system; SD: standard deviation.

**Table S3 Baseline characteristics of patients with DM who had disruption of SOPC visit by drug-refill service (N=** **13,960)**

| Characteristics | Overall | Drug-refill service (no, N=13,453) | Drug-refill service (yes, N=507) | P value |
| --- | --- | --- | --- | --- |
| Age (years) | 67.86±13.00 | 67.84±12.94 | 68.40±14.40 | 0.35 |
| > 65 years | 8,104 (58.05) | 7,811 (58.06) | 293 (57.79) | 0.90 |
| Sex, male | 6,599 (47.27) | 6,356 (47.25) | 243 (47.93) | 0.76 |
| CCI | 1.58±1.24 | 1.56±1.20 | 1.97±1.86 | <0.001 |
| ≥ 2 | 4,104 (29.40) | 3,904 (29.02) | 200 (39.45) | <0.001 |
| Disease duration (years) | 6.78 (3.60) | 6.75 (3.61) | 7.55 (3.31) | <0.001 |
| Smoker | 4,519 (32.37) | 4,370 (32.48) | 149 (29.39) | 0.14 |
| A&E attendance in 2020, yes | 2,668 (19.11) | 2,563 (19.05) | 105 (20.71) | 0.35 |
| Hospital admission in 2020, yes | 1,595 (11.43) | 1,513 (11.25) | 82 (16.17) | 0.001 |
| Primary care clinic attendance in 2020, yes | 10,299 (73.78) | 10,039 (74.62) | 260 (51.28) | <0.001 |
| Number of drug-refill service in 2020 | 0.06±0.36 | 0.00±0.00 | 1.55±1.13 | <0.001 |
| Number of primary care clinic attendance in 2020 | 2.92±2.58 | 2.96±2.59 | 1.75±2.07 | <0.001 |
| Clinical parameters |  |  |  |  |
| HbA1c (%) | 6.96±1.25 | 6.96±1.24 | 7.11±1.38 | 0.01 |
| SBP (mmHg) | 134.00±16.29 | 133.98±16.27 | 134.61±17.03 | 0.39 |
| DBP (mmHg) | 73.31±11.10 | 73.29±11.11 | 73.83±10.97 | 0.28 |
| eGFR (mL/min/1.73 m^2^) | 78.59±21.02 | 78.64±20.98 | 77.30±22.01 | 0.16 |
| UACR (mg/mmol) | 9.25±18.18 | 9.17±18.08 | 11.31±20.69 | 0.01 |
| BMI (kg/m^2^) | 25.71±4.48 | 25.71±4.47 | 25.90±4.74 | 0.34 |
| Lipid panel |  |  |  |  |
| LDL-C (mmol/L) | 2.15±0.69 | 2.15±0.69 | 2.15±0.71 | 0.98 |
| HDL-C (mmol/L) | 1.30±0.35 | 1.30±0.35 | 1.30±0.34 | 0.57 |
| Total cholesterol (mmol/L) | 4.14±0.84 | 4.14±0.84 | 4.15±0.86 | 0.71 |
| Triglyceride (mmol/L) | 1.53±1.00 | 1.52±0.98 | 1.61±1.33 | 0.28 |
| Medicine use | 6.96±1.25 | 6.96±1.24 | 7.11±1.38 | 0.01 |
| Insulin | 1,229 (8.80) | 1,130 (8.40) | 99 (19.53) | <0.001 |
| Oral antidiabetic drugs | 8,749 (62.67) | 8,438 (62.72) | 311 (61.34) | 0.53 |
| Lipid lowering drugs | 8,672 (62.12) | 8,332 (61.93) | 340 (67.06) | 0.02 |
| RAS blockers | 6,608 (47.34) | 6,342 (47.14) | 266 (52.47) | 0.02 |
| Beta blockers | 2,860 (20.49) | 2,750 (20.44) | 110 (21.70) | 0.49 |
| Other antihypertensive drugs | 7,530 (53.94) | 7,242 (53.83) | 288 (56.80) | 0.19 |
| Comorbidities |  |  |  |  |
| Hypertension | 10,875 (77.90) | 10,514 (78.15) | 361 (71.20) | <0.001 |

Data are presented in mean ± SD or number (percentage) as appropriate.

DM: diabetes mellitus; CCI: Charlson Comorbidity Index; A&E: accident and emergency; SOPC: specialist outpatient clinic; HbA1c: glycated hemoglobin; SBP: systolic blood pressure; DBP: diastolic blood pressure; eGFR: estimated glomerular filtration rate; BMI: body mass index; LDL-C: low-density lipoprotein cholesterol; HDL-C: high-density lipoprotein cholesterol; UACR: urinary albumin-to-creatinine ratio; RAS: renin-angiotensin-system; SD: standard deviation.

**Table S4 Data completion rates of the baseline characteristics among patients with DM (N=96,372)**

| **Clinical parameters** | N (%) |
| --- | --- |
|  |  |
| HbA1c (%) | 95,417 (99.0%) |
| SBP (mmHg) | 67,306 (69.8%) |
| DBP (mmHg) | 67,306 (69.8%) |
| eGFR (mL/min/1.73 m^2^) | 96,146 (99.8%) |
| UACR (mg/mmol) | 52,678 (54.7%) |
| BMI (kg/m^2^) | 79,423 (82.4%) |
| LDL-C (mmol/L) | 94,894 (98.5%) |
| HDL-C (mmol/L) | 95,003 (98.6%) |
| Total cholesterol (mmol/L) | 95,052 (98.6%) |
| Triglycerides (mmol/L) | 95,026 (98.6%) |
| Overall | 38,534 (40.0%) |

DM: diabetes mellitus; HbA1c: glycated hemoglobin; SBP: systolic blood pressure; DBP: diastotic blood pressure; eGFR: estimated glomerular filtration rate; UACR: urinary albumin-to-creatinine ratio; BMI: body mass index; LDL-C: low-density lipoprotein cholesterol; HDL-C: high-density lipoprotein cholesterol.

**Table S5 Data completion rate of the baseline characteristics among patients with DM who had disruption of SOPC visit (N=13,960)**

| **Clinical parameters** | N (%) |
| --- | --- |
|  |  |
| HbA1c (%) | 13,662 (97.9%) |
| SBP (mmHg) | 12,845 (92.0%) |
| DBP (mmHg) | 12,845 (92.0%) |
| eGFR (mL/min/1.73 m^2^) | 13,875 (99.4%) |
| UACR (mg/mmol) | 9,060 (64.9%) |
| BMI (kg/m^2^) | 12,216 (87.5%) |
| LDL-C (mmol/L) | 13,498 (96.7%) |
| HDL-C (mmol/L) | 13,523 (96.9%) |
| Total cholesterol (mmol/L) | 13,537 (97.0%) |
| Triglycerides (mmol/L) | 13,529 (96.9%) |
| Overall | 8,372 (60.0%) |

DM: diabetes mellitus; HbA1c: glycated hemoglobin; SBP: systolic blood pressure; DBP: diastotic blood pressure; eGFR: estimated glomerular filtration rate; UACR: urinary albumin-to-creatinine ratio; BMI: body mass index; LDL-C: low-density lipoprotein cholesterol; HDL-C: high-density lipoprotein cholesterol.

**Table S6 Association between disruption of SOPC visit and all-cause mortality, CVD and kidney failure incidence among patients with DM in sensitivity analysis using competing risk model (N=96,372)**

| **Outcomes** | **SOPC disruption (N=13,960)** | | | **SOPC attendance (Reference) (N=82,412)** | | | **Adjusted HR (95% CI)** |
| --- | --- | --- | --- | --- | --- | --- | --- |
|  | **Event** | **Person-month** | **Crude incidence rate (cases, 1000 person-month)** | **Event** | **Person-month** | **Crude incidence rate (cases, 1000 person-month)** |  |
| **CVD** | 1,476 | 554,778 | 2.66 (2.53,2.80) | 8,469 | 3,281,778 | 2.58 (2.53,2.64) | 1.09 (1.02,1.17)* |
| **CHD** | 714 | 571,740 | 1.25 (1.16,1.34) | 4,022 | 3,377,353 | 1.19 (1.15,1.23) | 1.05 (0.95,1.16) |
| **HF** | 327 | 579,779 | 0.56 (0.51,0.63) | 2,442 | 3,411,215 | 0.72 (0.69,0.74) | 1.15 (1.00,1.31)* |
| **Stroke** | 598 | 573,195 | 1.04 (0.96,1.13) | 3,171 | 3,394,897 | 0.93 (0.90,0.97) | 1.05 (0.94,1.17) |
| **Kidney failure** | 535 | 575,832 | 0.93 (0.85,1.01) | 5,217 | 3,351,680 | 1.56 (1.51,1.60) | 1.07 (0.97,1.19) |

Adjusted for age, sex, Charlson Comorbidity Index, smoking, disease duration, systolic blood pressure, diastolic blood pressure, hospital admission (yes or no), accident and emergency (yes or no), primary care clinic (yes or no), drug-refill service (yes or no), frequency of SOPC visits in 2020, frequency of primary care clinic visits in 2020, frequency of drug-refill service in 2020, glycated hemoglobin, estimated glomerular filtration rate, urinary albumin-to-creatinine ratio, body mass index, low-density lipoprotein cholesterol, high-density lipoprotein cholesterol, total cholesterol, triglycerides, insulin, oral antihyperglycemic drugs, lipid lowering drugs, renin-angiotensin-system blockers, beta blockers, other antihypertensive drugs and hypertension.

*: P< 0.05.

SOPC: specialist outpatient clinics; CVD: cardiovascular disease; CHD: coronary heart disease; HF: heart failure; DM: diabetes mellitus; HR: hazard ratio; CI: confidence interval.

**Table S7 Association between alternative modes of care and all-cause mortality, CVD and kidney failure incidence among patients with DM who had disruption of SOPC visit in sensitivity analysis using competing risk model (N=13,960)**

| **Outcomes** | **Alternative modes of care** | | | **Reference** | |  | **Adjusted HR (95% CI)** |
| --- | --- | --- | --- | --- | --- | --- | --- |
|  | **Event** | **Person-month** | **Crude incidence rate (cases, 1000 person-month)** | **Event** | **Person-month** | **Crude incidence rate (cases, 1000 person-month)** |  |
| **Primary care clinic** |  |  |  |  |  |  |  |
| CVD | 1,125 | 408,448 | 2.75 (2.60,2.92) | 351 | 146,330 | 2.40 (2.16,2.66) | 0.88 (0.75,1.03) |
| CHD | 554 | 421,064 | 1.32 (1.21,1.43) | 160 | 150,676 | 1.06 (0.91,1.24) | 0.90 (0.72,1.12) |
| HF | 228 | 427,994 | 0.53 (0.47,0.61) | 99 | 151,785 | 0.65 (0.54,0.79) | 0.69 (0.49,0.98)* |
| Stroke | 450 | 422,751 | 1.06 (0.97,1.17) | 148 | 150,444 | 0.98 (0.84,1.16) | 0.97 (0.76,1.25) |
| Kidney failure | 350 | 425,959 | 0.82 (0.74,0.91) | 185 | 149,873 | 1.23 (1.07,1.43) | 0.82 (0.62,1.08) |
| **Drug-refill** |  |  |  |  |  |  |  |
| CVD | 51 | 20,180 | 2.53 (1.92,3.33) | 1,425 | 534,598 | 2.67 (2.53,2.81) | 0.82 (0.50,1.34) |
| CHD | 22 | 20,859 | 1.05 (0.69,1.60) | 692 | 550,881 | 1.26 (1.17,1.35) | 1.27 (0.57,2.86) |
| HF | 14 | 20,986 | 0.67 (0.40,1.13) | 313 | 558,793 | 0.56 (0.50,0.63) | 0.80 (0.38,1.71) |
| Stroke | 22 | 20,762 | 1.06 (0.70,1.61) | 576 | 552,433 | 1.04 (0.96,1.13) | 0.65 (0.34,1.26) |
| Kidney failure | 28 | 20,781 | 1.35 (0.93,1.95) | 507 | 555,051 | 0.91 (0.84,1.00) | 1.09 (0.59,2.02) |

Adjusted for age, sex, Charlson Comorbidity Index, smoking, disease duration, systolic blood pressure, diastolic blood pressure, hospital admission (yes or no), accident and emergency (yes or no), primary care clinic (yes or no), drug-refill service (yes or no), frequency of primary care clinic visits in 2020, frequency of drug-refill service in 2020, glycated hemoglobin, estimated glomerular filtration rate, urinary albumin-to-creatinine ratio, body mass index, low-density lipoprotein cholesterol, high-density lipoprotein cholesterol, total cholesterol, triglycerides, insulin, oral antihyperglycemic drugs, lipid lowering drugs, renin-angiotensin-system blockers, beta blockers, other antihypertensive drugs and hypertension.

*: P< 0.05.

SOPC: specialist outpatient clinics; CVD: cardiovascular disease; CHD: coronary heart disease; HF: heart failure; DM: diabetes mellitus; HR: hazard ratio; CI: confidence interval.

**Table S8 Association between disruption of SOPC visit in 2020 and all-cause mortality, CVD and kidney failure incidence among patients with DM in sensitivity analysis of excluding patients with COVID-19 (N=70,558)**

| **Outcomes** | **SOPC disruption (N=10,437)** | | | **SOPC attendance (Reference) (N=60,121)** | | | **Adjusted HR (95% CI)** |
| --- | --- | --- | --- | --- | --- | --- | --- |
|  | **Event** | **Person-month** | **Crude incidence rate (cases, 1000 person-month)** | **Event** | **Person-month** | **Crude incidence rate (cases, 1000 person-month)** |  |
| **All-cause mortality** | 902 | 417,130 | 2.16 (2.03,2.31) | 5,972 | 2,391,629 | 2.50 (2.43,2.56) | 1.20 (1.11,1.30)*** |
| **CVD** | 973 | 417,188 | 2.33 (2.19,2.48) | 5,311 | 2,410,718 | 2.20 (2.14,2.26) | 1.14 (1.04,1.24)* |
| **CHD** | 472 | 428,407 | 1.10 (1.01,1.21) | 2,572 | 2,470,156 | 1.04 (1.00,1.08) | 1.06 (0.94,1.20) |
| **HF** | 211 | 433,967 | 0.49 (0.42,0.56) | 1,473 | 2,494,229 | 0.59 (0.56,0.62) | 1.17 (0.99,1.39) |
| **Stroke** | 387 | 429,812 | 0.90 (0.82,0.99) | 1,945 | 2,483,505 | 0.78 (0.75,0.82) | 1.14 (1.00,1.31) |
| **Kidney failure** | 336 | 431,386 | 0.78 (0.70,0.87) | 3,299 | 2,453,922 | 1.34 (1.30,1.39) | 1.06 (0.93,1.21) |

Adjusted for age, sex, Charlson Comorbidity Index, smoking, disease duration systolic blood pressure, diastolic blood pressure, hospital admission (yes or no), accident and emergency (yes or no), primary care clinic (yes or no), drug-refill service (yes or no), frequency of SOPC visits in 2020, frequency of primary care clinic visits in 2020, frequency of drug-refill service in 2020, glycated hemoglobin, estimated glomerular filtration rate, urinary albumin-to-creatinine ratio, body mass index, low-density lipoprotein cholesterol, high-density lipoprotein cholesterol, total cholesterol, triglycerides, insulin, oral antihyperglycemic drugs, lipid lowering drugs, renin-angiotensin-system blockers, beta blockers, other antihypertensive drugs and hypertension.

*: P< 0.05; ***: P< 0.001.

SOPC: specialist outpatient clinics; CVD: cardiovascular disease; CHD: coronary heart disease; HF: heart failure; DM: diabetes mellitus; HR: hazard ratio; CI: confidence interval.

**Table S9 Association between alternative modes of care and all-cause mortality, CVD and kidney failure incidence among patients with DM who had disruption of SOPC visit in 2020 in sensitivity analysis of excluding patients with COVID-19 (N=10,437)**

| **Outcomes** | **Alternative modes of care** | | | **Reference** | |  | **Adjusted HR (95% CI)** |
| --- | --- | --- | --- | --- | --- | --- | --- |
|  | **Event** | **Person-month** | **Crude incidence rate (cases, 1000 person-month)** | **Event** | **Person-month** | **Crude incidence rate (cases, 1000 person-month)** |  |
| **Primary care clinic** |  |  |  |  |  |  |  |
| All-cause mortality | 564 | 304,112 | 1.85 (1.71,2.01) | 338 | 113,018 | 2.99 (2.69,3.33) | 0.68 (0.54,0.87)** |
| CVD | 740 | 300,425 | 2.46 (2.29,2.65) | 233 | 116,763 | 2.00 (1.76,2.27) | 0.89 (0.72,1.10) |
| CHD | 370 | 308,694 | 1.20 (1.08,1.33) | 102 | 119,713 | 0.85 (0.70,1.03) | 0.95 (0.70,1.30) |
| HF | 153 | 313,412 | 0.49 (0.42,0.57) | 58 | 120,555 | 0.48 (0.37,0.62) | 0.71 (0.46,1.10) |
| Stroke | 283 | 310,419 | 0.91 (0.81,1.02) | 104 | 119,393 | 0.87 (0.72,1.06) | 0.92 (0.66,1.28) |
| Kidney failure | 220 | 312,264 | 0.70 (0.62,0.80) | 116 | 119,122 | 0.97 (0.81,1.17) | 0.83 (0.57,1.21) |
| **Drug-refill** |  |  |  |  |  |  |  |
| All-cause mortality | 57 | 14,252 | 4.00 (3.09,5.18) | 845 | 402,878 | 2.10 (1.96,2.24) | 1.16 (0.66,2.06) |
| CVD | 27 | 14,995 | 1.80 (1.23,2.63) | 946 | 402,193 | 2.35 (2.21,2.51) | 0.72 (0.35,1.45) |
| CHD | 9 | 15,426 | 0.58 (0.30,1.12) | 463 | 412,981 | 1.12 (1.02,1.23) | 0.83 (0.18,3.96) |
| HF | 6 | 15,462 | 0.39 (0.17,0.86) | 205 | 418,505 | 0.49 (0.43,0.56) | 0.56 (0.12,2.64) |
| Stroke | 13 | 15,247 | 0.85 (0.50,1.47) | 374 | 414,565 | 0.90 (0.82,1.00) | 0.68 (0.28,1.64) |
| Kidney failure | 16 | 15,240 | 1.05 (0.64,1.71) | 320 | 416,146 | 0.77 (0.69,0.86) | 1.78 (0.51,6.30) |

Adjusted for age, sex, Charlson Comorbidity Index, smoking, disease duration, systolic blood pressure, diastolic blood pressure, hospital admission (yes or no), accident and emergency (yes or no), primary care clinic (yes or no), drug-refill service (yes or no), frequency of primary care clinic visits in 2020, frequency of drug-refill service in 2020, glycated hemoglobin, estimated glomerular filtration rate, urinary albumin-to-creatinine ratio, body mass index, low-density lipoprotein cholesterol, high-density lipoprotein cholesterol, total cholesterol, triglycerides, insulin, oral antihyperglycemic drugs, lipid lowering drugs, renin-angiotensin-system blockers, beta blockers, other antihypertensive drugs and hypertension.

**: P< 0.01.

SOPC: specialist outpatient clinics; CVD: cardiovascular disease; CHD: coronary heart disease; HF: heart failure; DM: diabetes mellitus; HR: hazard ratio; CI: confidence interval.

**Table S10 Association between disruption of SOPC visit and all-cause mortality, CVD and kidney failure incidence among patients with DM in sensitivity analysis of including patients with at least 1 year of follow-up (N=** 90,414**)**

| **Outcomes** | **SOPC disruption (N=13,117)** | | | **SOPC attendance (Reference) (N=77,297)** | | | **Adjusted HR (95% CI)** |
| --- | --- | --- | --- | --- | --- | --- | --- |
|  | **Event** | **Person-month** | **Crude incidence rate (cases, 1000 person-month)** | **Event** | **Person-month** | **Crude incidence rate (cases, 1000 person-month)** |  |
| **All-cause mortality** | 851 | 537,862 | 1.58 (1.48,1.69) | 5,772 | 3,159,922 | 1.83 (1.78,1.87) | 1.12 (1.03,1.22)* |
| **CVD** | 973 | 537,212 | 1.81 (1.70,1.93) | 5,615 | 3,165,282 | 1.77 (1.73,1.82) | 1.10 (1.01,1.19)* |
| **CHD** | 481 | 544,321 | 0.88 (0.81,0.97) | 2,613 | 3,208,992 | 0.81 (0.78,0.85) | 1.08 (0.96,1.22) |
| **HF** | 219 | 547,790 | 0.40 (0.35,0.46) | 1,576 | 3,224,454 | 0.49 (0.47,0.51) | 1.20 (1.02,1.42)* |
| **Stroke** | 371 | 545,769 | 0.68 (0.61,0.75) | 2,077 | 3,216,801 | 0.65 (0.62,0.67) | 1.03 (0.90,1.19) |
| **Kidney failure** | 375 | 545,517 | 0.69 (0.62,0.76) | 3,541 | 3,192,872 | 1.11 (1.07,1.15) | 1.06 (0.94,1.20) |

Adjusted for age, sex, Charlson Comorbidity Index, smoking, disease duration systolic blood pressure, diastolic blood pressure, hospital admission (yes or no), accident and emergency (yes or no), primary care clinic (yes or no), drug-refill service (yes or no), frequency of SOPC visits in 2020, frequency of primary care clinic visits in 2020, frequency of drug-refill service in 2020, glycated hemoglobin, estimated glomerular filtration rate, urinary albumin-to-creatinine ratio, body mass index, low-density lipoprotein cholesterol, high-density lipoprotein cholesterol, total cholesterol, triglycerides, insulin, oral antihyperglycemic drugs, lipid lowering drugs, renin-angiotensin-system blockers, beta blockers, other antihypertensive drugs and hypertension.

*: P< 0.05.

SOPC: specialist outpatient clinics; CVD: cardiovascular disease; CHD: coronary heart disease; HF: heart failure; DM: diabetes mellitus; HR: hazard ratio; CI: confidence interval.

**Table S11 Association between alternative modes of care and all-cause mortality, CVD and kidney failure incidence among patients with DM who had disruption of SOPC visit in sensitivity analysis of including patients with at least 1 year of follow-up (N=13,117)**

| **Outcomes** | **Alternative modes of care** | | | **Reference** | |  | **Adjusted HR (95% CI)** |
| --- | --- | --- | --- | --- | --- | --- | --- |
|  | **Event** | **Person-month** | **Crude incidence rate (cases, 1000 person-month)** | **Event** | **Person-month** | **Crude incidence rate (cases, 1000 person-month)** |  |
| **Primary care clinic** |  |  |  |  |  |  |  |
| All-cause mortality | 558 | 399,787 | 1.40 (1.28,1.52) | 293 | 138,075 | 2.12 (1.89,2.38) | 0.75 (0.60,0.94)* |
| CVD | 738 | 397,743 | 1.86 (1.73,1.99) | 235 | 139,469 | 1.68 (1.48,1.91) | 0.80 (0.66,0.98)* |
| CHD | 363 | 403,195 | 0.90 (0.81,1.00) | 118 | 141,126 | 0.84 (0.70,1.00) | 0.77 (0.58,1.02) |
| HF | 153 | 405,804 | 0.38 (0.32,0.44) | 66 | 141,986 | 0.46 (0.37,0.59) | 0.71 (0.44,1.14) |
| Stroke | 284 | 404,123 | 0.70 (0.63,0.79) | 87 | 141,646 | 0.61 (0.50,0.76) | 0.96 (0.69,1.33) |
| Kidney failure | 251 | 404,542 | 0.62 (0.55,0.70) | 124 | 140,975 | 0.88 (0.74,1.05) | 0.88 (0.62,1.24) |
| **Drug-refill** |  |  |  |  |  |  |  |
| All-cause mortality | 59 | 18,793 | 3.14 (2.43,4.05) | 792 | 519,069 | 1.53 (1.42,1.64) | 0.94 (0.59,1.48) |
| CVD | 34 | 19,186 | 1.77 (1.27,2.48) | 939 | 518,026 | 1.81 (1.70,1.93) | 1.15 (0.58,2.27) |
| CHD | 17 | 19,441 | 0.87 (0.54,1.41) | 464 | 524,880 | 0.88 (0.81,0.97) | 1.37 (0.49,3.85) |
| HF | 11 | 19,535 | 0.56 (0.31,1.02) | 208 | 528,255 | 0.39 (0.34,0.45) | 0.96 (0.31,3.00) |
| Stroke | 10 | 19,616 | 0.51 (0.27,0.95) | 361 | 526,153 | 0.69 (0.62,0.76) | 0.72 (0.25,2.09) |
| Kidney failure | 21 | 19,454 | 1.08 (0.70,1.66) | 354 | 526,063 | 0.67 (0.61,0.75) | 1.18 (0.45,3.08) |

Adjusted for age, sex, Charlson Comorbidity Index, smoking, disease duration systolic blood pressure, diastolic blood pressure, hospital admission (yes or no), accident and emergency (yes or no), primary care clinic (yes or no), drug-refill service (yes or no), frequency of primary care clinic visits in 2020, frequency of drug-refill service in 2020, glycated hemoglobin, estimated glomerular filtration rate, urinary albumin-to-creatinine ratio, body mass index, low-density lipoprotein cholesterol, high-density lipoprotein cholesterol, total cholesterol, triglycerides, insulin, oral antihyperglycemic drugs, lipid lowering drugs, renin-angiotensin-system blockers, beta blockers, other antihypertensive drugs and hypertension.

*: P< 0.05.

SOPC: specialist outpatient clinics; CVD: cardiovascular disease; CHD: coronary heart disease; HF: heart failure; DM: diabetes mellitus; HR: hazard ratio; CI: confidence interval.

**Table 12 Association between disruption of SOPC visit and all-cause mortality, CVD and kidney failure incidence among patients with DM who had at least one SOPC attendance during the follow-up period (N=** **83,100)**

| **Outcomes** | **SOPC disruption (N=5,331)** | | | **SOPC attendance (Reference) (N=77,769)** | | | **Adjusted HR (95% CI)** |
| --- | --- | --- | --- | --- | --- | --- | --- |
|  | **Event** | **Person-month** | **Crude incidence rate % (cases, 1000 person-month)** | **Event** | **Person-month** | **Crude incidence rate % (cases, 1000 person-month)** |  |
| **All-cause mortality** | 448 | 216,680 | 2.07 (1.88,2.27) | 7,567 | 3,128,083 | 2.42 (2.37,2.47) | 1.11 (1.00,1.23)* |
| **CVD** | 950 | 198,675 | 4.78 (4.49,5.10) | 8,101 | 2,992,330 | 2.71 (2.65,2.77) | 1.73 (1.60,1.87)*** |
| **CHD** | 483 | 207,699 | 2.33 (2.13,2.54) | 3,846 | 3,062,354 | 1.26 (1.22,1.30) | 1.73 (1.54,1.93)*** |
| **HF** | 231 | 212,871 | 1.09 (0.95,1.23) | 2,371 | 3,093,210 | 0.77 (0.74,0.80) | 1.84 (1.58,2.14)*** |
| **Stroke** | 359 | 209,984 | 1.71 (1.54,1.90) | 3,021 | 3,079,718 | 0.98 (0.95,1.02) | 1.53 (1.34,1.74)*** |
| **Kidney failure** | 324 | 212,695 | 1.52 (1.37,1.70) | 5,004 | 3,063,142 | 1.63 (1.59,1.68) | 1.50 (1.32,1.70)*** |

Adjusted for age, sex, Charlson Comorbidity Index, smoking, disease duration, systolic blood pressure, diastolic blood pressure, hospital admission (yes or no), accident and emergency (yes or no), primary care clinic (yes or no), drug-refill service (yes or no), frequency of SOPC visits in 2020, frequency of primary care clinic visits in 2020, frequency of drug-refill service in 2020, glycated hemoglobin, estimated glomerular filtration rate, urinary albumin-to-creatinine ratio, body mass index, low-density lipoprotein cholesterol, high-density lipoprotein cholesterol, total cholesterol, triglycerides, insulin, oral antihyperglycemic drugs, lipid lowering drugs, renin-angiotensin-system blockers, beta blockers, other antihypertensive drugs and hypertension.

*: P< 0.05; ***: P< 0.001.

SOPC: specialist outpatient clinics; CVD: cardiovascular disease; CHD: coronary heart disease; HF: heart failure; DM: diabetes mellitus; HR: hazard ratio; CI: confidence interval.

**Table 13 Association between alternative modes of care and all-cause mortality, CVD and kidney failure incidence among patients with DM who had disruption of SOPC visit in 2020 but had at least one SOPC attendance during the follow-up period (N=5,331)**

| **Outcomes** | **Alternative modes of care** | | | **Reference** | |  | **Adjusted HR (95% CI)** |
| --- | --- | --- | --- | --- | --- | --- | --- |
|  | **Event** | **Person-month** | **Crude incidence rate % (cases, 1000 person-month)** | **Event** | **Person-month** | **Crude incidence rate % (cases, 1000 person-month)** |  |
| **Primary care clinic** |  |  |  |  |  |  |  |
| All-cause mortality | 315 | 162,277 | 1.94 (1.74,2.17) | 133 | 54,403 | 2.44 (2.06,2.90) | 0.91 (0.67,1.23) |
| CVD | 733 | 148,225 | 4.95 (4.60,5.32) | 217 | 50,450 | 4.30 (3.77,4.91) | 0.81 (0.66,1.01) |
| CHD | 376 | 155,047 | 2.43 (2.19,2.68) | 107 | 52,652 | 2.03 (1.68,2.46) | 0.85 (0.63,1.15) |
| HF | 165 | 159,583 | 1.03 (0.89,1.20) | 66 | 53,288 | 1.24 (0.97,1.58) | 0.60 (0.39,0.91)* |
| Stroke | 276 | 157,158 | 1.76 (1.56,1.98) | 83 | 52,826 | 1.57 (1.27,1.95) | 0.92 (0.65,1.31) |
| Kidney failure | 226 | 159,484 | 1.42 (1.24,1.61) | 98 | 53,211 | 1.84 (1.51,2.24) | 0.96 (0.67,1.39) |
| **Drug-refill** |  |  |  |  |  |  |  |
| All-cause mortality | 46 | 12,524 | 3.67 (2.75,4.90) | 402 | 204,156 | 1.97 (1.79,2.17) | 1.02 (0.61,1.70) |
| CVD | 43 | 11,797 | 3.64 (2.70,4.91) | 907 | 186,878 | 4.85 (4.55,5.18) | 0.74 (0.41,1.34) |
| CHD | 20 | 12,219 | 1.64 (1.06,2.54) | 463 | 195,480 | 2.37 (2.16,2.59) | 1.11 (0.40,3.06) |
| HF | 12 | 12,321 | 0.97 (0.55,1.71) | 219 | 200,550 | 1.09 (0.96,1.25) | 0.61 (0.21,1.77) |
| Stroke | 18 | 12,226 | 1.47 (0.93,2.34) | 341 | 197,758 | 1.72 (1.55,1.92) | 0.66 (0.29,1.52) |
| Kidney failure | 22 | 12,317 | 1.79 (1.18,2.71) | 302 | 200,378 | 1.51 (1.35,1.69) | 0.96 (0.44,2.11) |

Adjusted for age, sex, Charlson Comorbidity Index, smoking, disease duration, systolic blood pressure, diastolic blood pressure, hospital admission (yes or no), accident and emergency (yes or no), primary care clinic (yes or no), drug-refill service (yes or no), frequency of primary care clinic visits in 2020, frequency of drug-refill service in 2020, glycated hemoglobin, estimated glomerular filtration rate, urinary albumin-to-creatinine ratio, body mass index, low-density lipoprotein cholesterol, high-density lipoprotein cholesterol, total cholesterol, triglycerides, insulin, oral antihyperglycemic drugs, lipid lowering drugs, renin-angiotensin-system blockers, beta blockers, other antihypertensive drugs and hypertension.

*: P< 0.05.

SOPC: specialist outpatient clinics; CVD: cardiovascular disease; CHD: coronary heart disease; HF: heart failure; DM: diabetes mellitus; HR: hazard ratio; CI: confidence interval.

**Table 14 Association between disruption of SOPC visit and all-cause mortality, CVD and kidney failure incidence among patients with DM in sensitivity analysis using fine stratification weighting (N=** **96,372)**

| **Outcomes** | **SOPC disruption (N=13,960)** | | | **SOPC attendance (Reference) (N=82,412)** | | | **Adjusted HR (95% CI)** |
| --- | --- | --- | --- | --- | --- | --- | --- |
|  | **Event** | **Person-month** | **Crude incidence rate % (cases, 1000 person-month)** | **Event** | **Person-month** | **Crude incidence rate % (cases, 1000 person-month)** |  |
| **All-cause mortality** | 2,185 | 535,449 | 4.08 (3.76,4.43) | 8,391 | 3,295,781 | 2.55 (2.49,2.60) | 1.60 (1.53,1.68)*** |
| **CVD** | 1,642 | 508,362 | 3.23 (2.97,3.52) | 8,483 | 3,153,726 | 2.69 (2.63,2.75) | 1.20 (1.14,1.27)* |
| **CHD** | 733 | 523,926 | 1.40 (1.24,1.59) | 4,055 | 3,226,129 | 1.26 (1.22,1.30) | 1.11 (1.03,1.21)** |
| **HF** | 463 | 528,736 | 0.88 (0.74,1.05) | 2,376 | 3,261,408 | 0.73 (0.70,0.76) | 1.20 (1.09,1.33)*** |
| **Stroke** | 690 | 524,115 | 1.32 (1.15,1.51) | 3,188 | 3,244,786 | 0.98 (0.95,1.02) | 1.34 (1.23,1.45)*** |
| **Kidney failure** | 881 | 526,388 | 1.67 (1.47,1.91) | 4,946 | 3,234,494 | 1.53 (1.49,1.57) | 1.09 (1.02,1.18)* |

Hazard ratios were adjusted for age, sex, Charlson Comorbidity Index, smoking, disease duration, systolic blood pressure, diastolic blood pressure, hospital admission (yes or no), accident and emergency (yes or no), primary care clinic (yes or no), drug-refill service (yes or no), frequency of primary care clinic visits in 2020, frequency of drug-refill service in 2020, glycated hemoglobin, estimated glomerular filtration rate, urinary albumin-to-creatinine ratio, body mass index, low-density lipoprotein cholesterol, high-density lipoprotein cholesterol, total cholesterol, triglycerides, insulin, oral antihyperglycemic drugs, lipid lowering drugs, renin-angiotensin-system blockers, beta blockers, other antihypertensive drugs, hypertension and weighting.

*: P< 0.05; **: P< 0.01; ***: P< 0.001.

SOPC: specialist outpatient clinics; CVD: cardiovascular disease; CHD: coronary heart disease; HF: heart failure; DM: diabetes mellitus; HR: hazard ratio; CI: confidence interval.

**Table 15 Association between alternative modes of care and all-cause mortality, CVD and kidney failure incidence among patients with DM who had disruption of SOPC visit in sensitivity analysis using fine stratification weighting (N=13,960)**

| **Outcomes** | **Alternative modes of care** | | | **Reference** | |  | **Adjusted HR (95% CI)** |
| --- | --- | --- | --- | --- | --- | --- | --- |
|  | **Event** | **Person-month** | **Crude incidence rate % (cases, 1000 person-month)** | **Event** | **Person-month** | **Crude incidence rate % (cases, 1000 person-month)** |  |
| **Primary care clinic** |  |  |  |  |  |  |  |
| All-cause mortality | 972 | 412,583 | 2.36 (2.14,2.60) | 569 | 140,969 | 4.04 (3.59,4.55) | 0.58 (0.53,0.65)*** |
| CVD | 1,111 | 392,305 | 2.83 (2.63,3.06) | 416 | 134,802 | 3.09 (2.68,3.57) | 0.92 (0.82,1.03) |
| CHD | 534 | 403,172 | 1.33 (1.19,1.48) | 188 | 138,405 | 1.36 (1.09,1.70) | 0.98 (0.83,1.15) |
| HF | 240 | 409,192 | 0.59 (0.49,0.71) | 119 | 139,304 | 0.86 (0.66,1.12) | 0.69 (0.55,0.86)*** |
| Stroke | 467 | 403,828 | 1.16 (1.02,1.32) | 164 | 138,595 | 1.19 (0.95,1.49) | 0.98 (0.82,1.17) |
| Kidney failure | 434 | 407,953 | 1.06 (0.90,1.26) | 200 | 139,172 | 1.44 (1.19,1.76) | 0.74 (0.62,0.87)*** |
| **Drug-refill** |  |  |  |  |  |  |  |
| All-cause mortality | 65 | 19,597 | 3.33 (2.45,4.61) | 1,233 | 539,069 | 2.29 (2.16,2.42) | 1.25 (0.97,1.61) |
| CVD | 43 | 18,910 | 2.27 (1.54,3.49) | 1,427 | 513,863 | 2.78 (2.64,2.93) | 0.76 (0.56,1.03) |
| CHD | 22 | 19,310 | 1.14 (0.61,2.36) | 692 | 526,864 | 1.31 (1.22,1.42) | 0.83 (0.54,1.26) |
| HF | 10 | 19,468 | 0.51 (0.25,1.23) | 316 | 534,517 | 0.59 (0.53,0.66) | 0.80 (0.42,1.50) |
| Stroke | 15 | 19,290 | 0.77 (0.44,1.44) | 576 | 528,993 | 1.09 (1.00,1.18) | 0.65 (0.39,1.09) |
| Kidney failure | 26 | 19,391 | 1.37 (0.75,2.78) | 514 | 533,918 | 0.96 (0.88,1.05) | 1.30 (0.88,1.92) |

Hazard ratios were adjusted for age, sex, Charlson Comorbidity Index, smoking, disease duration, systolic blood pressure, diastolic blood pressure, hospital admission (yes or no), accident and emergency (yes or no), primary care clinic (yes or no), drug-refill service (yes or no), frequency of primary care clinic visits in 2020, frequency of drug-refill service in 2020, glycated hemoglobin, estimated glomerular filtration rate, urinary albumin-to-creatinine ratio, body mass index, low-density lipoprotein cholesterol, high-density lipoprotein cholesterol, total cholesterol, triglycerides, insulin, oral antihyperglycemic drugs, lipid lowering drugs, renin-angiotensin-system blockers, beta blockers, other antihypertensive drugs, hypertension and weighting.

***: P< 0.001.

SOPC: specialist outpatient clinics; CVD: cardiovascular disease; CHD: coronary heart disease; HF: heart failure; DM: diabetes mellitus; HR: hazard ratio; CI: confidence interval.
